# Removal of Arsenate From Groundwater by Cathode of Bioelectrochemical System Through Microbial Electrosorption, Reduction, and Sulfuration

**DOI:** 10.3389/fmicb.2022.812991

**Published:** 2022-03-11

**Authors:** Honghong Yuan, Yumeng Huang, Ouyuan Jiang, Yue Huang, Dongsheng Qiu, Williamson Gustave, Xianjin Tang, Zhongjian Li

**Affiliations:** ^1^Zhejiang Provincial Key Laboratory of Agricultural Resources and Environment, College of Environmental and Resource Sciences, Institute of Soil and Water Resources and Environmental Science, Zhejiang University, Hangzhou, China; ^2^ZJU-Hangzhou Global Scientific and Technological Innovation Center, Hangzhou, China; ^3^Key Laboratory of Biomass Chemical Engineering of Ministry of Education, Zhejiang University, Hangzhou, China; ^4^School of Chemistry, Environmental and Life Sciences, University of The Bahamas, Nassau, Bahamas; ^5^Institute of Zhejiang University - Quzhou, Quzhou, China

**Keywords:** arsenate removal, bioelectrochemical system, microbial electrosorption, microbial reduction, sulfuration

## Abstract

Arsenate [As(V)] is a toxic metalloid and has been observed at high concentrations in groundwater globally. In this study, a bioelectrochemical system (BES) was used to efficiently remove As(V) from groundwater, and the mechanisms involved were systematically investigated. Our results showed that As(V) can be efficiently removed in the BES cathode chamber. When a constant cell current of 30 mA (*I*_*cell*_, volume current density = 66.7 A/m^3^) was applied, 90 ± 3% of total As was removed at neutral pH (7.20–7.50). However, when *I*_*cell*_ was absent, the total As in the effluent, mainly As(V), had increased approximately 2–3 times of the As(V) in influent. In the abiotic control reactor, under the same condition, no significant total As or As(V) removal was observed. These results suggest that As(V) removal was mainly ascribed to microbial electrosorption of As(V) in sludge. Moreover, part of As(V) was bioelectrochemically reduced to As(III), and sulfate was also reduced to sulfides [S(–II)] in sludge. The XANES results revealed that the produced As(III) reacted with S(–II) to form As_2_S_3_, and the residual As(III) was microbially electroadsorbed in sludge. This BES-based technology requires no organic or chemical additive and has a high As(V) removal efficiency, making it an environment-friendly technique for the remediation of As-contaminated groundwater.

## Introduction

Arsenic (As) contamination in groundwater possesses a serious threat to human and ecosystem health globally due to the high toxicity and ubiquity of As (Rodriguez-Freire et al., 2016; Wang et al., 2020). As in groundwater originates from the natural weathering, dissolution of As-bearing minerals (Smedley and Kinniburgh, 2002; Garelick et al., 2008), and anthropogenic activities such as the semiconductor industry, alloy manufacturing, and agricultural production (Mandal and Suzuki, 2002; Liu et al., 2015; Peng et al., 2018). Groundwater constitutes the largest reservoir of drinking water. Hence, drinking water has been identified as a major dietary source of inorganic As. Studies have shown that As pollution can lead to numerous diseases such as cancers, circulatory disorders, hypertension, neurological impairment, and reproductive disorders (Navas-Acien et al., 2019; Zheng, 2020). The maximum permissible As concentration limit proposed by the World Health Organization (WHO, 2017) and the US Environmental Protection Agency (US-EPA, 2001) is 10 μg L^– 1^ in the drinking water. However, more than 137 million people in over 70 countries have been exposed to a wide range of As in drinking water (Rahman et al., 2009; McArthur et al., 2016; Schwanck et al., 2016). Therefore, there is an urgent need to develop methods that can efficiently remove As from groundwater to produce safe drinking water and prevent imminent health catastrophes.

In groundwater, As is found predominately in the forms of arsenite [As(III)] and arsenate [As(V)] (Smedley and Kinniburgh, 2002; Abid et al., 2016). As(III) is present as non-ionic species, arsenious acid (H_3_AsO_3_) at near neutral pH (5–8) under reducing conditions in deep groundwater (Welch et al., 2000; Sorg et al., 2014). On the other hand, As(V) mainly exists as oxy-anionic species (HAsO_4_^2–^ and H_2_AsO_4_^–^) under oxidizing conditions and is typically found in shallow groundwater (Welch et al., 2000). Generally, As(III) is easily oxidized to As(V) (Zhou et al., 2013). Thus, the oxidization of As(III) to As(V) as a pretreatment has been regarded as an efficient approach to remove As(III) (Nidheesh et al., 2020; Wang et al., 2020). However, treatments for As decontamination of shallow groundwater are focused on As(V) removal (Chen et al., 2018) given that As(V) dominates As species in shallow ground water. To date, various methods have been developed to remove As(V) from aqueous solution (Zakhar et al., 2018). These methods include chemical precipitation (Peng et al., 2018), coagulation (Mohora et al., 2018), adsorption (Xiong et al., 2017; Ajith et al., 2019), ion exchange (Donia et al., 2011), and membrane filtration (Ungureanu et al., 2015). Nevertheless, the current methods have some drawbacks such as high costs associated with chemical amendments, catalysts application, and generation of secondary pollutants (Zakhar et al., 2018). Therefore, it is necessary to develop novel, efficient, and eco-friendly methods for As(V) removal from groundwater.

Bioelectrochemical system (BES) has been developed as a promising biotechnology for wastewater treatment (Qiu et al., 2017; Ai et al., 2020; Wan et al., 2020). In BESs, microorganisms can exchange electrons with solid electrodes to carry out oxidation and reduction reactions (Sydow et al., 2014; Logan et al., 2019). For example, biocathode can serve as an electron donor and provides a reducing environment for removing heavy metal(loid) ions in aqueous solution (Tandukar et al., 2009; Qin et al., 2012). Sulfate naturally occurs in groundwater, and sulfate reduction can be facilitated by sulfate-reducing bacteria (SRB) in the biocathode of the BES using electrode or hydrogen as electron donors (Hu et al., 2019). Moreover, numerous studies have shown that As(V) can be removed via the formation of orpiment (As_2_S_3_) by the combination of microbial reduction of As(V) to As(III) and the microbial reduction of sulfate to sulfides [S(–II)] under reducing conditions (Chung et al., 2006; Rodriguez-Freire et al., 2016). In addition to forming precipitation, some studies found that As(V) can also be removed by microbial biosorption (Zhu et al., 2018; Naveed et al., 2020; Zhang et al., 2020). For instance, a study showed that extracellular polymeric substances (EPSs) secreted by *Bacillus* can form complexes with inorganic As and facilitate As removal via electrostatic attraction to a BES cathode (Wang et al., 2021). Therefore, the mechanism behind As(V) removal from groundwater by the BES cathode needs to be elucidated in the presence of sulfate.

In this work, a BES was constructed to investigate the feasibility of using the biocathode for As(V) removal from groundwater. The BES required long-term operation to achieve efficient As(V) removal from the contaminated groundwater. Moreover, the underlying mechanism of As(V) removal was elucidated by exploring the roles of electricity, microbes, and S(–II). Furthermore, the effect of sulfate on As(V) removal was also evaluated. Overall, this study developed a potential and promising technology for efficient As(V) removal from As-contaminated groundwater.

## Materials and Methods

### Bioelectrochemical System Construction and Operation

**FIGURE 1:**
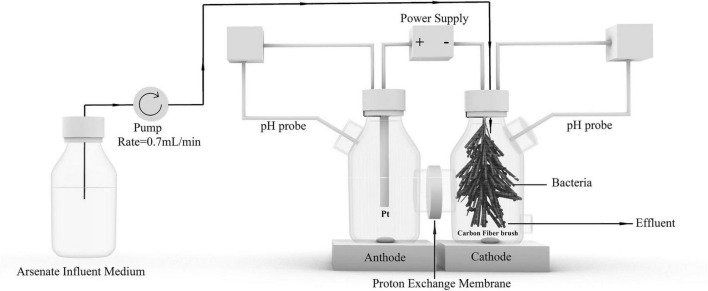
Schematic diagram of the BES for arsenate removal from groundwater.

Platinum (Pt) mesh and carbon fiber brush were used as the anode and cathode in a double-chambered BES, respectively. The anode and cathode were prepared as described in a previous study (Blazquez et al., 2017). The separator was a proton exchange membrane (19.6 cm^2^, Nafion 117, DuPont Company, United States). The membrane was pretreated in 50 mM phosphate buffer solution overnight. The carbon fiber brush (8 cm in length, 3 cm in diameter) cathode in a 500-mL cathode chamber was used to conduct experiments. The schematic diagram of the BES used in this study is shown in Figure 1.

The BES cathode chamber was inoculated with 50-mL sludge suspension from an anaerobic bioreactor, which was originally inoculated with a bacterial consortium obtained from As-contaminated river sediments at a mining site (Liuyang, China, 113°31′ 12.69″E, 28°13′38.55″N) and Qige sewage treatment plant (Hangzhou, China). The anaerobic bioreactor was operated for 180 days before the inoculum was sampled. The content of suspended solids in sludge suspension was 0.042 g/g wet weight. The influent of the cathode chamber contains buffer solution (NaHCO_3_ 1.00 g L^– 1^, NH_4_Cl 0.30 g L^– 1^, KCl 0.10 g L^– 1^, MgCl_2_⋅6H_2_O 0.13 g L^– 1^, K_2_HPO_4_⋅3H_2_O 3.79 g L^– 1^, KH_2_PO_4_ 2.12 g L^– 1^), 5 mL L^– 1^ trace minerals, and 2.5 mL L^– 1^ vitamin solution (Blazquez et al., 2017). Amounts of 100 μM As(V) as Na_2_HAsO_4_⋅7H_2_O and 3.3 mM sulfate as Na_2_SO_4_ were added to the cathode medium to acclimate the bacteria that are able to reduce As(V) and sulfate. The medium in the anode chamber contains buffer solution (Na_2_HPO_4_⋅12H_2_O 46.2 g L^– 1^, NaH_2_PO_4_⋅2H_2_O 11.1 g L^– 1^). The BES was operated in galvanostatic mode, and the currents (*I*_*cell*_) were controlled at 30 mA. The constant *I*_*cell*_ was applied to the electrodes using a potentiostat (VSP-300, Biologic Co., France). Ag/AgCl electrode (in saturated KCl) was used as a reference electrode for measuring cathode potentials. The BES was operated in a continuous flow model. The flow rate was 0.70 ml min^– 1^, and the hydraulic retention time (HRT) was 12 h. The pH was adjusted to a near neutral range (7.2–7.5) by adding NaOH or HCl using a pH modulator in both chambers. In addition, an abiotic control reactor was also constructed, and the cathode chamber of that reactor was not inoculated with the sludge suspension.

### Experimental Design

The As(V) removal was explored in the BES cathode chamber. During the operation, 100 μM As(V) and 3.3 mM Na_2_SO_4_ were added to the influent, and a constant *I*_*cell*_ of 30 mA was applied to the electrodes. The influent and effluent samples were collected daily. The collected samples were filtered through 0.22-μm syringe filters to measure the concentrations of the total As, As(III), As(V), and dissolved S(–II).

To uncover the mechanisms of As(V) removal in the BES cathode chamber, the roles of electricity, microbes, S(–II), and sulfate were investigated. (i) To determine the role of electricity, an *I*_*cell*_ of 30 mA was applied or absent to the BES and the abiotic control reactor. The effects of *I*_*cell*_ intensity (30, 20, and 10 mA) on As(V) removal were also explored. About 100 or 1,000 μM As(V) and 3.3 mM Na_2_SO_4_ were added to the influent. (ii) To determine the role of microbes, the BES and the abiotic control reactor were operated with an *I*_*cell*_ of 30 mA. About 100 μM As(V) and 3.3 mM Na_2_SO_4_ were added to the influent. (iii) To determine the role of S(–II), 100 μM As(V) or As(III) and 3.3 mM Na_2_S were added to the influent of the abiotic control reactor, which was operated with and without an *I*_*cell*_ of 30 mA. Additionally, (iv) to determine the effect of sulfate on As(V) removal, the BES was operated with or without sulfate with an *I*_*cell*_ of 30 mA. About 100 μM As(V) was added to the influent. All experiments were carried out in triplicates. The influent and effluent samples were collected daily. All samples were filtered through 0.22-μm syringe filters to analyze the concentrations of total As, As(III), As(V), and dissolved S(–II). At day 71, the sludge suspension was sampled from the BES cathode chamber and centrifuged for 1 min at 10,000 *g*. The supernatant was used to measure total As, As(III), and As(V) concentrations, and the pellet was stored at –20°C.

### Chemical Analyses

Total As concentration was determined by the inductively coupled plasma mass spectrometry (ICP-MS, NEXION300XX, Perkin Elmer, United States). As(III) and As(V) concentrations were qualitatively identified by HPLC-ICP-MS as described in a previous study (Yuan et al., 2021). The sulfate concentrations were measured using an ion chromatograph equipped with an Ionpac AS19 analytical column (ICS-1100, Dionex, United States). Dissolved S(–II) concentration was analyzed by the iodometric method (Wu et al., 2020). The sludge samples were characterized using a scanning electron microscopy (SEM) and K-edge X-ray absorption spectra (XAS) with X-ray absorption near-edge structure (XANES) according to the methodology described in Supplementary Information.

### Microbial Analyses

DNA was extracted from the 0.5 g of the sludge sample using the Fast-DNA SPIN kit for soil (MP Biomedicals, United States) following the manufacturer’s instructions. PCR amplifications of the arsenate reductase gene (*arrA*, *arsC*), the sulfate reductase gene (*dsrA*, *dsrB*), and *16S* rRNA genes were performed with the primers As1F/As1R, amlt-42-F/amlt-376-R, Dsr1F + /Dsr-R, DSRp2060F/DSR4R, and 1369F/1492R, respectively. Details of primers are listed in Supplementary Table 1. The abundances of the *arrA*, *arsC*, *dsrA*, *dsrB*, and *16S* rRNA genes were determined in the sludge samples using the primers described above by quantitative real-time polymerase chain reaction (qPCR) performed on a StepOnePlus™ real-time PCR system (ABI, United States). Details of the qPCR are shown in Supplementary Information. The absolute gene copy numbers of *arrA*, *arsC*, *dsrA*, and *dsrB* were normalized to that of the ambient *16S* rRNA genes to limit the variation caused by the DNA extraction process, analytical method, and background microbial abundance (Zhang et al., 2017).

Moreover, the sludge samples were sequenced to analyze the functional microbial community structure. The *arsC*, *dsrA*, and *dsrB* amplicons were obtained with the same primers used for qPCR. The *arrA* gene was not amplified in the sludge sample; therefore, no further related information concerning that gene is described here. The obtained amplicons were sequenced on an Illumina MiSeq PE300 platform at the Majorbio Bio-Pharm Co., Ltd., Shanghai, China. Operational taxonomic units (OTUs) with 97% similarity cutoff (Edgar, 2013) were clustered using UPARSE (version 7.1) (Edgar, 2013), and chimeric sequences were identified and removed. The taxonomy of each OTU representative sequence was analyzed by the RDP Classifier (version 2.2) (Wang et al., 2007) against the NCBI database using a 0.7 confidence threshold. The sequences obtained in this study have been deposited in the NCBI Sequence Read Archive under the following accession numbers: *arsC* gene from SRR13357137, *dsrA* gene from SRR13357138, and *dsrB* gene from SRR13357136.

## Results and Discussion

### As(V) Removal Performance in the Bioelectrochemical System Cathode Chamber

**FIGURE 2:**
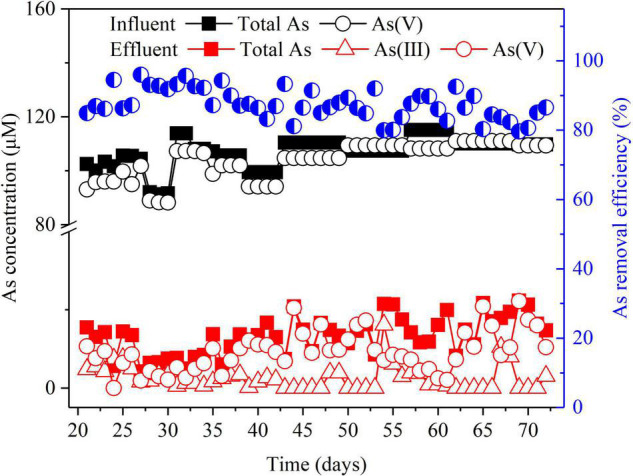
As removal in the BES cathode chamber. The left *Y*-axis indicates the concentrations of total As, As(V), and As(III) in the influent and the effluent, and the right *Y-*axis indicates total As removal efficiency in the effluent. Experimental conditions: influent [As(V)] = 100 μM, (Na_2_SO_4_) = 3.3 mM, *I*_*cell*_ = 30 mA.

To evaluate the overall As (V) removal efficiency, the BES was operated in galvanostatic mode with an *I*_*cell*_ of 30 mA. Figure 2 shows that during the long-term operation, 90 ± 3% of total As was removed in the BES cathode chamber. Various other technologies have been used for As(V) removal, and their As removal efficiencies range from 86 to 99% (Supplementary Table 2). Compared to these technologies, our BES cathode can achieve comparable high removal efficiency of As(V). Moreover, As(III) was detected in the effluent (Figure 2). This suggests that As(V) was partially converted to As(III) in the BES cathode chamber. S(–II) was also detected in the effluent (Supplementary Figure 1). Furthermore, if this BES technology is applied to the treatment of As-contaminated groundwater, the residual As can be removed through adsorption and membrane technology (Alka et al., 2021). Residual S(–II) can be further removed by aerated oxidation (Zhang et al., 2018) or electrochemically oxidized by the BES anode (Wu et al., 2020).

### The Underlying Mechanism of As(V) Removal in the Bioelectrochemical System Cathode Chamber

#### Microbial Electrosorption Dominates in As(V) Removal

**FIGURE 3:**
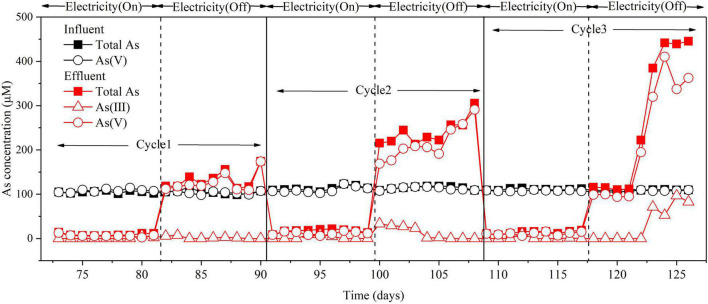
Effect of electricity on As(V) removal in the BES cathode chamber. Experimental conditions: influent [As(V)] = 100 μM, (Na_2_SO_4_) = 3.3 mM, *I*_*cell*_ = 30 mA. “On” indicates the *I*_*cell*_ was applied; “Off” indicates the *I*_*cell*_ was absent. Cycle 1, Cycle 2, and Cycle 3 represent three replicates of electricity conditions.

The role of electricity in As(V) removal in the BES cathode chamber was investigated. Firstly, the effect of *I*_*cell*_ intensity on As(V) removal was investigated. When *I*_*cell*_ was controlled at 30, 20, and 10 mA, the corresponding cathode potentials ranged from –1.15 to –1.25 V, –1.05 to–1.13 V, and –1.00 to –1.05 V, and the As removal efficiencies were 93–96%, 87–95%, and < 5%, respectively (Supplementary Figure 2). These results demonstrate that *I*_*cell*_ played a critical role in As(V) removal with the BES cathode. To further investigate the As(V) removal mechanism by the cathode, the electricity was switched between “On” (*I*_*cell*_ = 30 mA) and “Off” status for three successive cycles. When an *I*_*cell*_ of 30 mA was applied, the total As and As(V) were significantly decreased in the effluent. When *I*_*cell*_ was absent, the total As and As(V) in the effluent were significantly increased up to two to three times of the As(V) in the influent (Figure 3). It is noteworthy to mention that As(V) accounted for the majority of As released from the sludge when the *I*_*cell*_ was absent. This implies that microbial adsorption or mineral/electrode adsorption of As(V) was responsible for the As(V) removal in the BES cathode chamber (Miran et al., 2017).

To further reveal the role of microbes in As(V) removal, the electricity experiments were conducted in an abiotic control reactor. No total As or As(V) removal was observed with an *I*_*cell*_ of 30 mA, and no As(V) was released when *I*_*cell*_ was absent (Supplementary Figure 3). This result indicates that abiotic cathode electrodes could not adsorb As(V), even when the *I*_*cell*_ was applied. In addition, since no Fe-compound or Mn-compound was added to the influent during operation, the As(V) removal was not related to mineral adsorption. Thus, As(V) removal was mainly attributed to microbial electrosorption, which suggests that As(V) was adsorbed by microbes in the sludge attached on the carbon fiber brush electrode when electricity was input. According to the microbial electrosorption mechanism, As laden sludge is also produced during the operation. Hence, the separation of adsorbed As from the sludge will be a major issue for the practical application of this process (Tandukar et al., 2009).

Numerous studies have found that microorganisms have a high biosorption capacity for heavy metals by their cells or EPSs (Miran et al., 2017; Song et al., 2018). For example, studies showed that As was bound to functional groups such as C–O–C, C–O–H, C = O, –NH, and –OH in the EPSs through surface complexation or hydrophobic interactions (Zhu et al., 2018; Naveed et al., 2020). Moreover, As was reported to be adsorbed by positively charged EPSs through electrostatic interactions (Liu and Fang, 2002). The current intensity alters the composition of functional groups on the surface of EPSs, leading to the change of EPS charges (Sun et al., 2019). This explains the dependence of As(V) removal on the applied *I*_*cell*_.

#### Sulfuration Eliminates As(III) Produced From Microbial Reduction of As(V)

When an *I*_*cell*_ of 30 mA was applied, As(III) and S(–II) were produced in the BES cathode chamber (Figure 3, Supplementary Figure 4, and Supplementary Table 3). But no As(III) and S(–II) were produced in the effluent of the abiotic control reactor (Supplementary Figures 3, 4). This result suggests that bioelectrochemical reduction was responsible for the conversion of As(V) to As(III) and SO_4_^2–^ to S(–II). To further determine the specific microbes involved in the reduction of As(V) and sulfate, SEM, qPCR, and sequencing of the As(V) and sulfate-related functional genes were conducted for the sludge samples. The SEM analysis illustrates that some rod-shaped bacteria were presented in the sludge of the BES cathode chamber (Supplementary Figure 5). Subsequently, the relative gene abundances of the *arsC*, *arrA*, *dsrA*, and *dsrB* genes (normalized to the *16S* rRNA gene) were quantified for the sludge (Supplementary Figure 6). The results demonstrate that the As(V)-reducing bacteria and SRB coexist in the sludge of the BES cathode chamber. Furthermore, the microbial taxonomic composition of As(V)-reducing bacteria and SRB was identified in the sludge (Supplementary Figure 7 and Supplementary Table 4). At the genus level, *Shinella* (80.2%) was the major genus identified in the As(V)-reducing bacteria containing *arsC*. *Desulfotomaculum* (97.7%) was the major genus identified in the SRB containing *dsrB*, while the major genera (95.3%) containing *dsrA* were unclassified.

**FIGURE 4:**
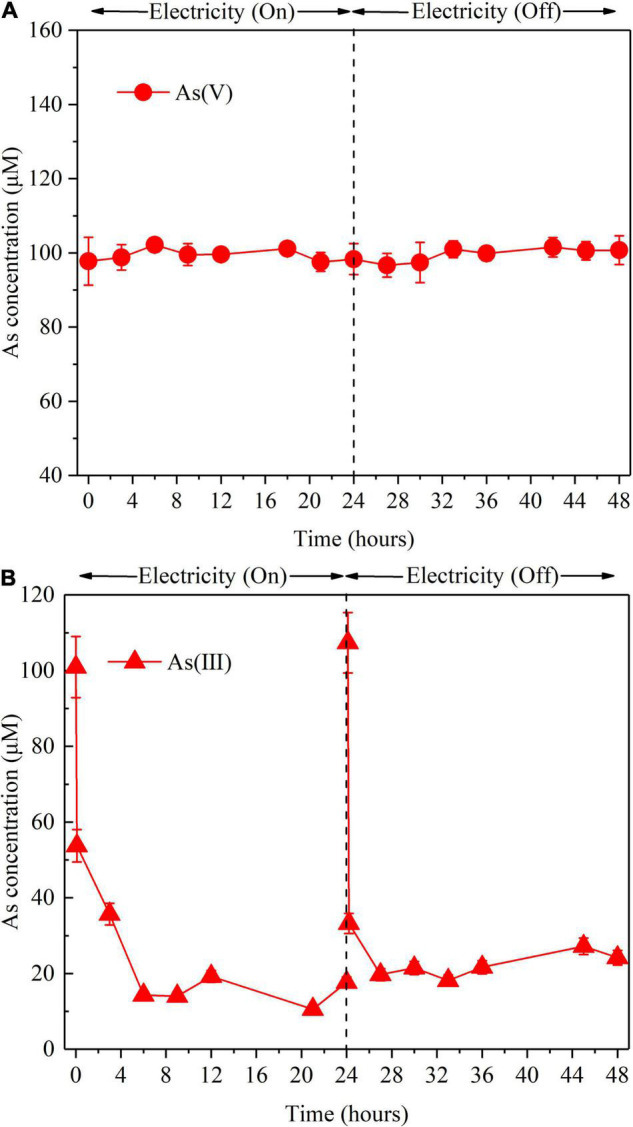
Effect of S(–II) on As removal in the abiotic control reactor with the influent containing As(V) **(A)** and containing As(III) **(B)** with applied *I*_*cell*_ or not, respectively. Experimental conditions: influent [As(V)] = 100 μM, [As(III)] = 100 μM, (Na_2_S) = 3.3 mM, *I*_*cell*_ = 30 mA. “On” indicates *I*_*cell*_ was applied; “Off” indicates the *I*_*cell*_ was absent. The error bars show the standard deviation (*n* = 3).

To clarify the role of S(–II) produced from sulfate reduction in As removal, the reactions between As(V) or As(V) and S(–II) were investigated in the abiotic control reactor (Figures 4A,B). When an *I*_*cell*_ of 30 mA was applied, As(V) did not react with S(–II) (Figure 4A), while As(III) can react with S(–II) (Figure 4B), which was consistent with previous studies (Rodriguez-Freire et al., 2014; Kong et al., 2017). Due to much higher S(–II) concentration than that of produced As(III), residual S(–II) was examined in the effluent of the BES cathode chamber (Supplementary Figure 4). Moreover, the As(III) produced during the As(V) removal process is likely to be precipitated from the effluent as As_2_S_3_. Therefore, S(–II) originated from bioelectrochemical reduction of sulfate played an important role in As removal in terms of eliminating As(III). Surprisingly, As(V) was detected in the abiotic control reactor, when the influent contained only As(III) (Supplementary Figure 8). This indicates that As(III) can be oxidized to As(V) in the abiotic control reactor by O_2_•^–^ or reactive chlorine species (HOCl or Cl•) produced during the course of the electrolysis (Kim et al., 2014).

#### Proposed Mechanism of As(V) Removal Verified by X-Ray Absorption Near-Edge Structure

**FIGURE 5:**
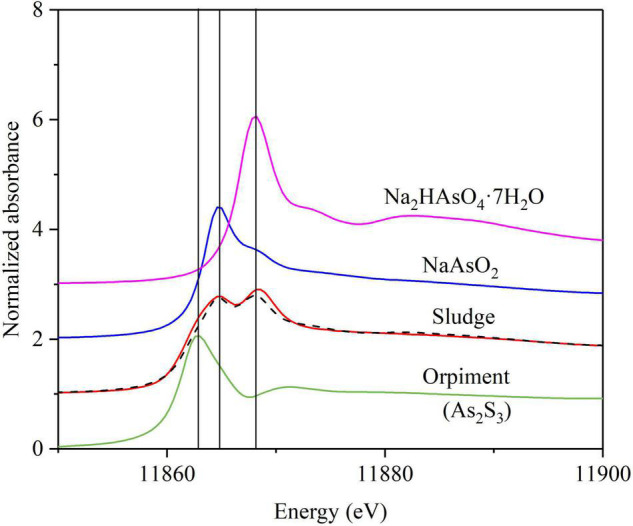
Arsenic K-α X-ray absorption near edge structure spectra (XANES) from the sludge in the BES cathode chamber. The XANES spectra (red solid lines) for solid phase fit (black dashed lines) by least-squares linear combination to standards of Na_2_HAsO_4_⋅7H_2_O [As (V), purple solid lines], NaAsO_3_ [As(III)] (blue solid lines), and orpiment (As_2_S_3_) (green solid lines); vertical lines indicate the diagnostic As species position (± 1 eV): 11,863 = arsenic sulfide; 11,864.6 = As(III); 11,868.2 = As (V). Fits and reported error are given in Supplementary Table 5.

The As speciation in the sludge of the BES cathode chamber was further investigated to verify the As(V) removal mechanism using XANES spectra. Figure 5 shows the XANES spectra for the sludge sampled from the BES cathode chamber along with those of Na_2_HAsO_4_⋅7H_2_O [As(V)], NaAsO_3_ [As(III)], and As_2_S_3_ references. The XANES fits showed that the As speciation in the sludge was 24.2% As(V), 45.0% As(V), and 33.0% As_2_S_3_ (Figure 5 and Supplementary Table 5). This result demonstrates that As(V) removal in the BES cathode chamber was divided in three pathways: (1) microbial electrosorption of As(V), (2) microbial reduction of As(V) to As(III) and consequently microbial electrosorption of As(III), and (3) microbial reduction of As(V) to As(III) and SO_4_^2–^ to S(–II) and the subsequent formation of the As_2_S_3_ precipitate. The main XANES peak absorbance for As(V) was shifted to a slightly higher energy compared to the reference curve (Supplementary Figure 8).

### Effect of Sulfate on As(V) Removal in the Bioelectrochemical System Cathode Chamber

**FIGURE 6:**
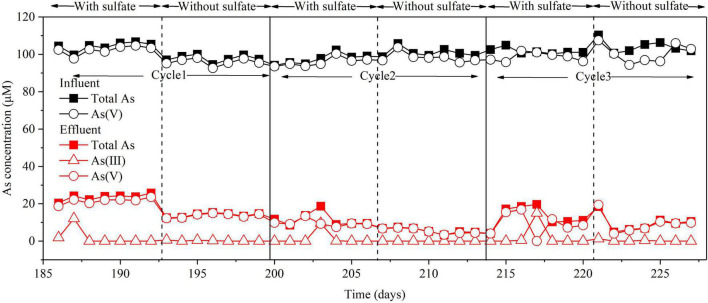
Effect of sulfate on As(V) removal in the BES cathode chamber. Experimental conditions: influent [As(V)] = 100 μM, (Na_2_SO_4_) = 3.3 mM, *I*_*cell*_ = 30 mA. Cycle 1, Cycle 2, and Cycle 3 represent three replicates of with or without sulfate.

In the BES, when *I*_*cell*_ was applied, H_2_ produced by H_2_O electrolysis could be used as an electron donor in two pathways: one to reduce As(V) to As(III) and the other one to reduce SO_4_^2–^ to S(–II) (Figure 2 and Supplementary Figure 1). Previous works have shown that sulfate reduction inhibited microbial As(V) reduction due to electron competition (Chung et al., 2006). Hence, the effect of sulfate on As(V) removal was explored (Figure 6). The results showed that similar removal efficiencies of total As and As(V) were achieved (about 90 and 93%) with or without the addition of sulfate in the influent. This suggests that sulfate does not affect As(V) removal in the BES cathode chamber. Based on the above results (Figures 3, 4B), the microbial electrosorption dominated in As removal, while As_2_S_3_ formation only accounted for a small proportion. Therefore, when no sulfate was added in the influent, S (–II) was not produced, and As (III) could not be removed by As_2_S_3_ formation. However, the removal efficiency of total As was not affected when no sulfate was added. To evaluate the efficiency of electron utilization in the BES cathode chamber, the coulombic efficiency (CE) was calculated based on sulfate reduction (Supplementary Equation 1). The CE at 30 mA was 50.57%. However, the CE based on As reduction cannot be calculated because of As(III) adsorption in the sludge. Due to the higher redox potential of SO_4_^2–^/HS^–^ (0.197 V) than As(V)/As(III) (–0.71 V) and the higher concentration of SO_4_^2–^ than that of As(V), sulfate is easier to be reduced than As(V). Thereby, the CE based on As reduction is expected to be lower than that based on sulfate reduction. By using H_2_ as the electron donor for sulfate reduction and As(V) reduction, the BES cathode does not need any organics as the electron donor and a continuous supply of sulfide reagents (Tandukar et al., 2009).

## Conclusion

This work demonstrates that As(V) could be efficiently removed in the BES cathode chamber. During the BES operation, the removal efficiency of total As was 90 ± 3% at neutral pH conditions. When an *I*_*cell*_ of 30 mA was applied, As(V) was significantly removed by microbial electrosorption in the sludge. Moreover, As(V)-reducing bacteria and SRB were identified in the sludge. Furthermore, during the As removal process, some of the As(V) was reduced to As(III) by the autotrophic As(V)-reducing bacteria. Subsequently, the by-product As(III) precipitated with S(–II) derived from the bioelectrochemical reduction of sulfate by the H_2_ autotrophic SRB. Simultaneously, As(III) can be electroadsorbed by microbial consortium in the sludge. Moreover, sulfate in groundwater did not affect the As(V) removal by the BES cathode. Furthermore, our BES cathode does not need organics as electron donors and a continuous supply of sulfide reagents. Therefore, it could become a potential and promising treatment technology for the As(V) removal from As-contaminated groundwater.

## Data Availability Statement

The original contributions presented in the study are included in the article/Supplementary Material, further inquiries can be directed to the corresponding author/s.

## Author Contributions

XT and ZL designed and supervised the project. HY, YmH, OJ, YeH, and DQ performed the experiments. HY analyzed the data and drafted the manuscript. WG, XT, and ZL revised the manuscript. All authors approved the final version for submission.

## Conflict of Interest

The authors declare that the research was conducted in the absence of any commercial or financial relationships that could be construed as a potential conflict of interest.

## Publisher’s Note

All claims expressed in this article are solely those of the authors and do not necessarily represent those of their affiliated organizations, or those of the publisher, the editors and the reviewers. Any product that may be evaluated in this article, or claim that may be made by its manufacturer, is not guaranteed or endorsed by the publisher.
