# Supplementary material for: Removal of Arsenate From Groundwater by Cathode of Bioelectrochemical System Through Microbial Electrosorption, Reduction, and Sulfuration
Source: Front Microbiol. 2022 Mar 11;13:812991. doi: 10.3389/fmicb.2022.812991 (PMC8963459; doi:10.3389/fmicb.2022.812991)
Supplement: Supplementary file 1 [file Data_Sheet_1.docx]

**Removal of arsenate from groundwater by cathode of bioelectrochemical system through microbial electrosorption, reduction and sulfuration**

Honghong Yuan^1,2†^, Yumeng Huang^3†^, Ouyuan Jiang^1^, Yue Huang^1^, Dongsheng Qiu^1^, Williamson Gustave^4^, Xianjin Tang^1^* and Zhongjian Li^3, 5^*

^1^ Institute of Soil and Water Resources and Environmental Science, College of Environmental and Resource Sciences, Zhejiang Provincial Key Laboratory of Agricultural Resources and Environment, Zhejiang University, Hangzhou, China

^2^ ZJU-Hangzhou Global Scientific and Technological Innovation Center, Hangzhou, China

^3^ College of Chemical and Biological Engineering, Key Laboratory of Biomass Chemical Engineering of Ministry of Education, Zhejiang University, Hangzhou, China

^4^ School of Chemistry, Environmental & Life Sciences, University of The Bahamas, New Providence, Nassau, Bahamas

^5^ Institute of Zhejiang University- Quzhou, Quzhou, China

*** Correspondence:**

Xianjin Tang

xianjin@zju.edu.cn

Zhongjian Li

zdlizj@zju.edu.cn

The supplementary information contains 13 pages with 8 figures and 5 tables.

**Supplementary Information**

**Materials and methods**

Solid phase characterization

Scanning Electron Microscopy (SEM) was performed using a GeminiSEM 300 (Carl Zeiss Microscopy GmbH, Germany) equipped with a field emission electron gun. The samples were vacuum dry and crushed to a powder material. Then, the samples were adhered to a metallic base and coated with platinum. Arsenic K-edge X-ray absorption spectra (XAS) were acquired at the Stanford Synchrotron Radiation Lightsource (SSRL) on beamline 11-2 under dedicated conditions (3 GeV, 300 mA) using an unfocused beam detuned 40% to remove high energy harmonics. The monochromator was calibrated to an As^0^ foil, with the edge jump inflection defined as 11867 eV. Samples for XAS were not vacuum dried, but were loaded as wet pastes into 0.5 mm thick aluminum sample holders, sealed with kapton tape in an anaerobic glovebag (Coy, MI), and kept under N_2_ until the time of analysis. To avoid post experiment and X-ray beam induced redox changes, all XAS data were collected in a LN_2_ cryostat (77 K), with the sample position moved at least 1 mm (always greater than the X-ray beam size) between scans to expose a fresh cross-sectional area of sample to the X-ray beam for X-ray absorption near-edge structure (XANES). Collected spectra (3-5 scans) were averaged in SixPack and the background was subtracted using a linear fit through the pre-edge region. Normalized XANES spectra were fit by least squares linear combination fits to model spectra of orpiment and realgar using the Athena package in Demeter software suite. Fit error and goodness of fit are reported with R-factor.

Extraction of DNA and quantitative PCR

DNA in the sludge was extracted from 0.5 g of samples using the Fast-DNA SPIN kit for soil (MP Biomedicals, USA) according to the manufacturer’s instructions. The DNA was dissolved in 100 μL sterilized deionized water and stored at -20 ℃ before use. The extracted DNA concentrations were measured photometrically using a Nanodrop 2000 spectrophotometer (Thermo Scientific, USA). The DNA from the collected samples were diluted with Ultra-Pure TM DNase/RNase-Free distilled water (Thermo-Fisher Scientific, USA) by ten-folds and subjected to real-time quantitative PCR (RT-qPCR) to determine the abundance of bacterial *arrA*, *arsC*, *dsrA*, *dsrB* and 16S rRNA genes in the sludge sample. The qPCR amplification and quantification were performed on a StepOnePlus™ real-time PCR system (ABI, USA) in 20 μL reaction mixture containing 10 μL of SYBR Premix Ex Taq® II (Takara, Japan), 0.4 μL of each 10 μM primers, 1.0 μL of template DNA, and 8.2 μL of ddH_2_O. The following PCR protocol was followed: 30 s at 95 °C, followed by 40 cycles of 15 s at 95 °C, 20 s at the respective annealing temperature, an extension for 30 s at 72 °C, then a final melt curve stage with temperature ramping from 60 °C to 95 °C. PCR amplifications of *arrA*, *arsC*, *dsrA*, *dsrB* and 16S rRNA genes were performed with the primers As1F/As1R (Zhang et al., 2015), amlt-42-F/amlt-376-R (Sun et al., 2014), Dsr1F+/Dsr-R (Kondo et al., 2014), DSRp2060F/DSR4R (Geets et al., 2006) and 1369F/1492R (Suzuki et al., 2000), respectively. Details of primers are listed in Table S1.

Coulombic efficiency (CE) calculations

$\text{CE}\text{=}\frac{\frac{\left( \text{C}_{\text{1}}\text{-}\text{C}_{\text{0}} \right)\text{×V}}{\text{M}}\text{×}\text{n}_{\text{e}}\text{×}\text{N}_{\text{A}}}{\text{I}_{\text{cell}}\text{×t}}$ (1)

*C_0_*—the substance concentration in influent,

*C_1_*—the substance concentration in effluent,

V—the wastewater volume,

M—the molar mass of the substance,

*I_cell_*—the input current in the electrodes,

t—the operation time of reactor,

*n_e_*—the number of electrons transferred

*N_A_*—the avogadro constant (6.02×10^23^).

**Table S1** The primers in the experiments.

| Target gene | Primer | Sequence (5’-3’) | Literature citation |
| --- | --- | --- | --- |
| *arsC* | amLt-42-F | TCGCGTAATACGCTGGAGAT | Sun et al., 2004 |
|  | amLt-376-R | ACTTTCTCGCCGTCTTCCTT |  |
| *arrA* | As1F | CGAAGTTCGTCCCGATHACNTGG | Zhang et al., 2015 |
|  | As1R | GGGGTGCGGTCYTTNARYTC |  |
| *dsrA* | Dsr 1F+ | ACSCACTGGAAGCACGGCGG | Kondo et al., 2004 |
|  | Dsr-R | GTGGMRCCGTGCAKRTTGG |  |
| *dsrB* | DSRp2060F | CAACATCGTYCAYACCCAGGG | Geets et al., 2006 |
|  | DSR4R | GTGTAGCAGTTACCGCA |  |
| *16S rRNA* | 1369F | CGGTGAATACGTTCYCGG | Suzuki et al., 2000 |
|  | 1492R | GGWTACCTTGTTACGACTT |  |

**Table S2** Summary of methods used to remove arsenic from water medium.

| Methods | Types | Removal efficiency | Arsenic concentration (μg L^-1^) | References |
| --- | --- | --- | --- | --- |
| Adsorption | Granular activated carbon | 99%  pH (1.3) | 500 | Zhang et al., 2007 |
|  |  |  |  |  |
| Ion Exchange | Synthetic resins | 98%  pH (2.0-7.0) | 100 | Karakurt et al, 2019 |
|  |  |  |  |  |
| Chemical precipitation | Fe(III)-precipitates | 95%  pH (7.0) | 525 | Senn et al., 2018 |
|  |  |  |  |  |
| Electro-  coagulation | Stainless steel electrode | 86%-99%  pH (5.2) | 10000-100000 | Gilhotra et al., 2018 |
|  |  |  |  |  |
| Membrane Technology | Nano-filtration | 97%  pH (7.0) | 100 | Ungureanu et al., 2015 |
|  |  |  |  |  |
| Microbial electrosorption | Bioelectrochemical Technology | 93-96%  pH (7.2-7.5) | 7500/75000 | This study |

**Table S3** Concentration of total As, As(Ⅲ) and As(Ⅴ) in the sludge supernatant of the BES cathode chamber.

| Sampling Time | Total As (μg/L) | As(Ⅲ) (μg/L) | As(Ⅴ) (μg/L) |
| --- | --- | --- | --- |
| Day 71 | 3119.20 ±23.41 | 1613.67 ±21.84 | 910.06 ±19.23 |

Mean values ± standard errors are shown (n = 3).

**Table S4** The proportion of reads in each genus for As(Ⅴ)-reducing bacteria encoded by *arsC* gene and sulfate-reducing bacteria (SRB) encoded by *dsrA* gene and *dsrB* gene in the sludge of the BES cathode chamber.

| As(Ⅴ)-reducing bacteria  (*arsC*) | | Sulfate-reducing bacteria  (*dsrA)* | | Sulfate-reducing bacteria  (*dsrB*) | |
| --- | --- | --- | --- | --- | --- |
| Genus | Percentage | Genus | Percentage | Genus | Percentage |
| *Agrobacterium* | 0.07% | *Achromobacter* | 0.03% | *Desulfotomaculum* | 97.73% |
| *Bordetella* | 1.18% | *Deinococcus* | 0.08% | *Simplicispira* | 0.03% |
| *Bradyrhizobium* | 0.29% | *Desulfotomaculum* | 3.35% | *Unclassified* | 2.24% |
| *Hoeflea* | 0.04% | *Dyella* | 0.09% |  |  |
| *Mesorhizobium* | 0.16% | *Nitrobacter* | 0.10% |  |  |
| *Microvirga* | 1.13% | *Oligotropha* | 0.11% |  |  |
| *Ochrobactrum* | 0.07% | *Paracoccus_f__Rhodobacteraceae* | 0.16% |  |  |
| *Oligotropha* | 1.63% | *Pseudomonas* | 0.03% |  |  |
| *Pandoraea* | 0.02% | *Rhodanobacter* | 0.07% |  |  |
| *Pannonibacter* | 1.73% | *Rhodopseudomonas* | 0.15% |  |  |
| *Paracoccus_f__Rhodobacteraceae* | 1.65% | *Simplicispira* | 0.24% |  |  |
| *Shinella* | 80.20% | *Sphaerotilus* | 0.16% |  |  |
| *Thauera* | 0.07% | *Starkeya* | 0.02% |  |  |
| *Variovorax* | 4.37% | *Thermomonas* | 0.03% |  |  |
| *Unclassified* | 7.39% | *Thiobacillus* | 0.04% |  |  |
|  |  | *Xanthomonas* | 0.02% |  |  |
|  |  | *Unclassified* | 95.33% |  |  |

**Table S5** The XANES fit results for Na_2_HAsO_4·_7H_2_O (As(V)), NaAsO_3_ (As(Ⅲ)) and orpiment (As_2_S_3_) in the sludge of the BES cathode chamber.

|  | Sample | Na_2_HAsO_4_·7H_2_O | NaAsO_3_ | As_2_S_3_ | R-factor |
| --- | --- | --- | --- | --- | --- |
| XANES | Sludge | 0.242 ± 0.007 | 0.450 ± 0.013 | 0.330± 0.010 | 0.0053 |

Mean values ± standard errors are shown (n = 3).


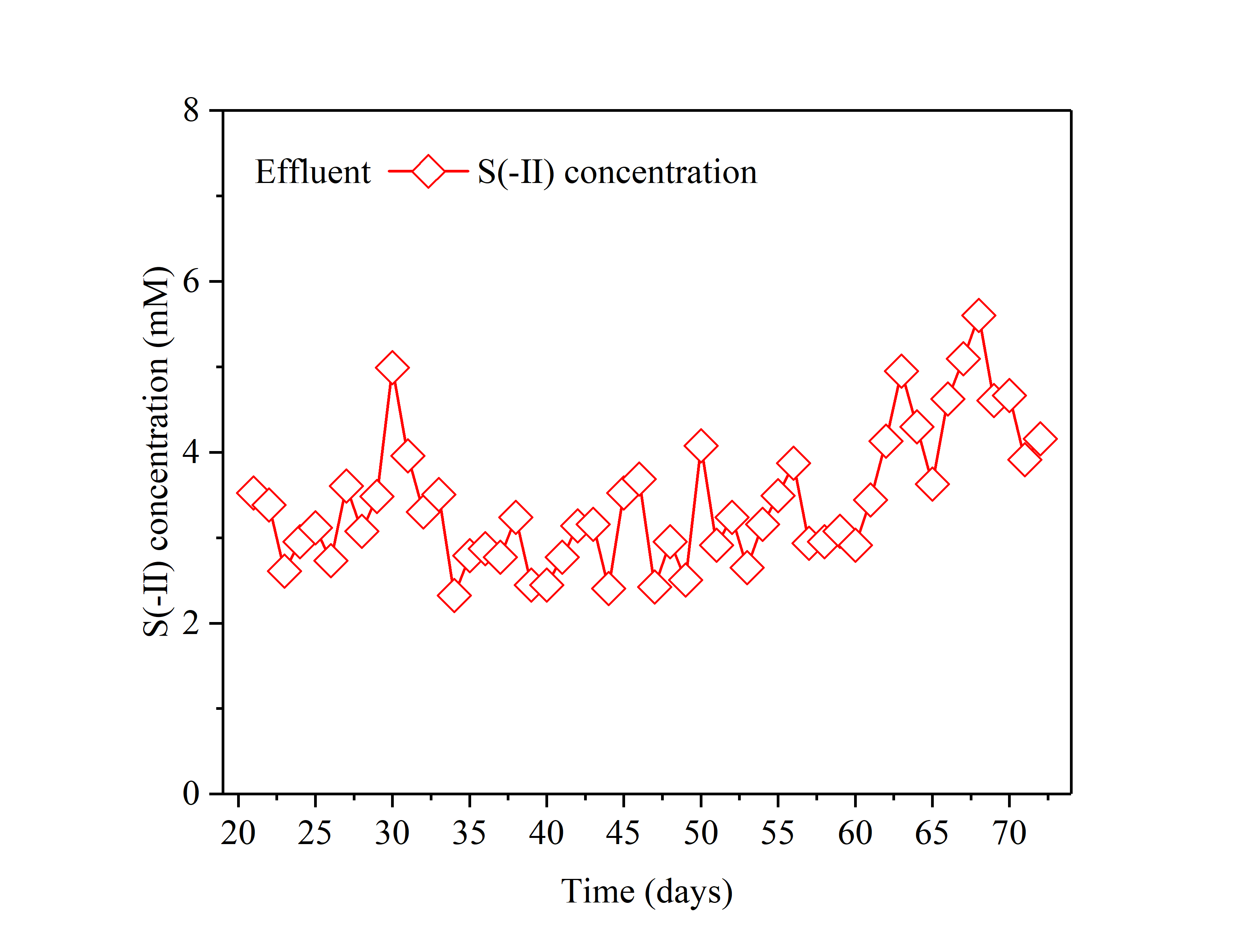


**Figure S1.** S(−Ⅱ) concentrations in effluent of the BES cathode chamber. Experimental conditions: Influent [As(Ⅴ)] = 100 μM, [Na_2_SO_4_] = 3.3 mM, *I_cell_* = 30 mA.


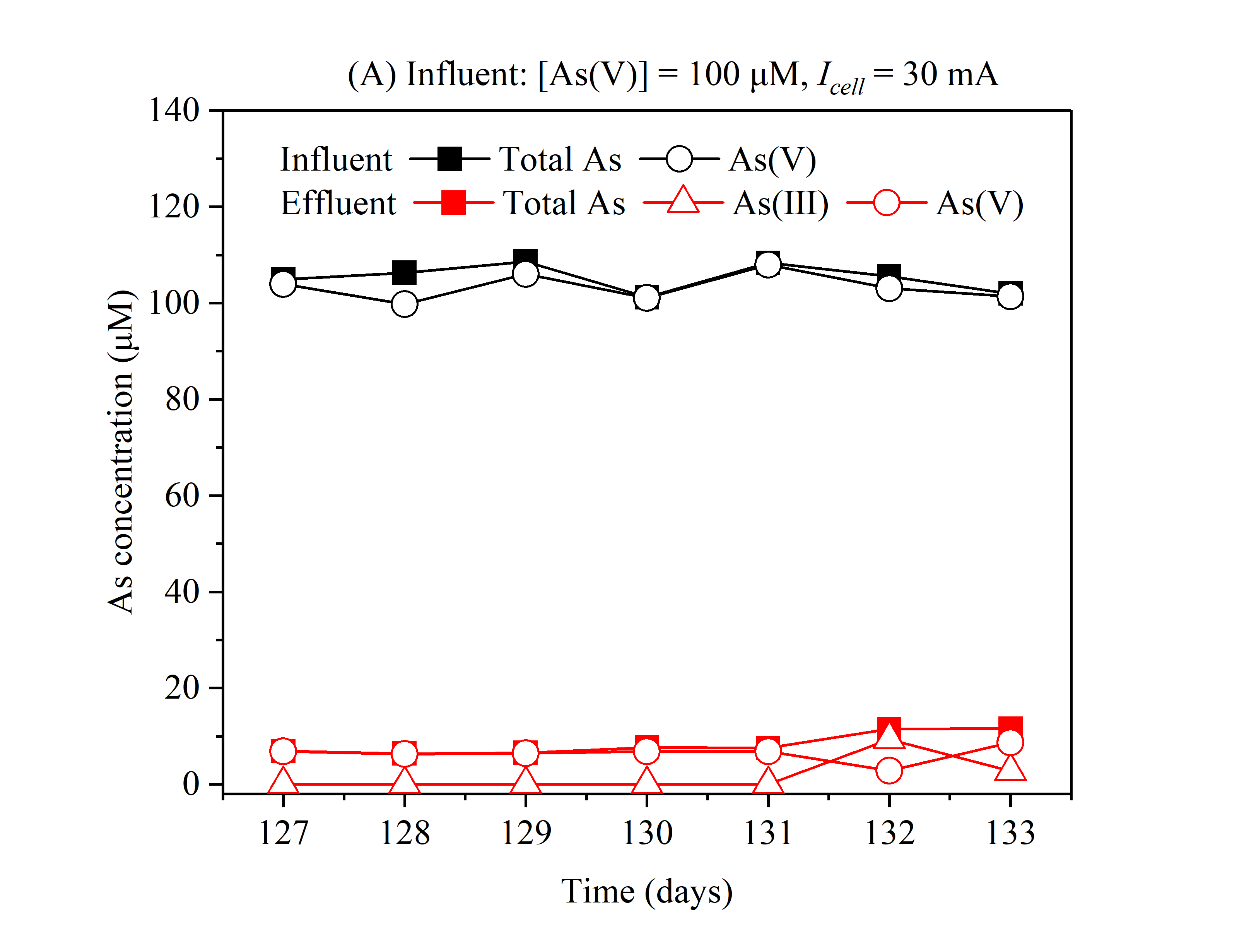

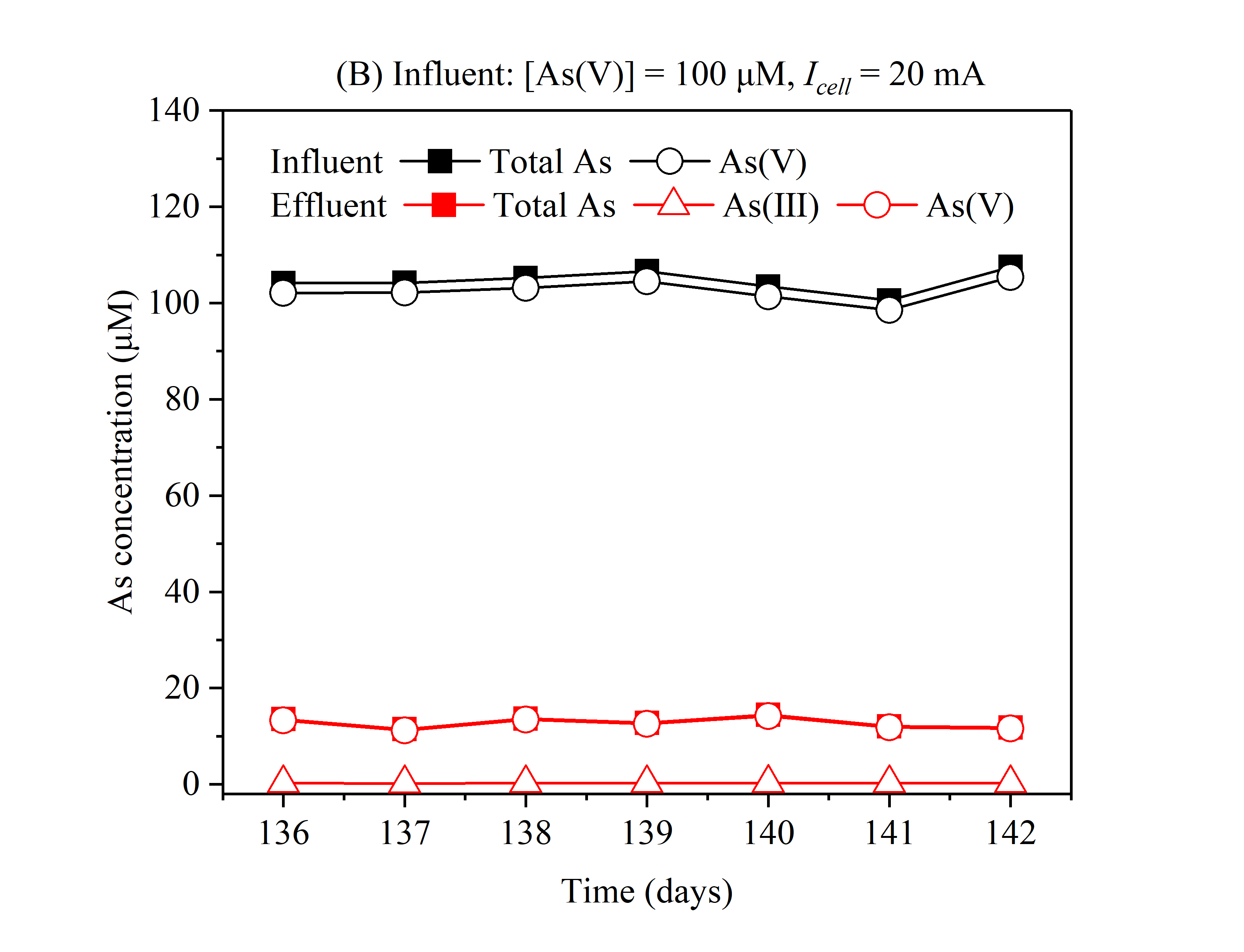

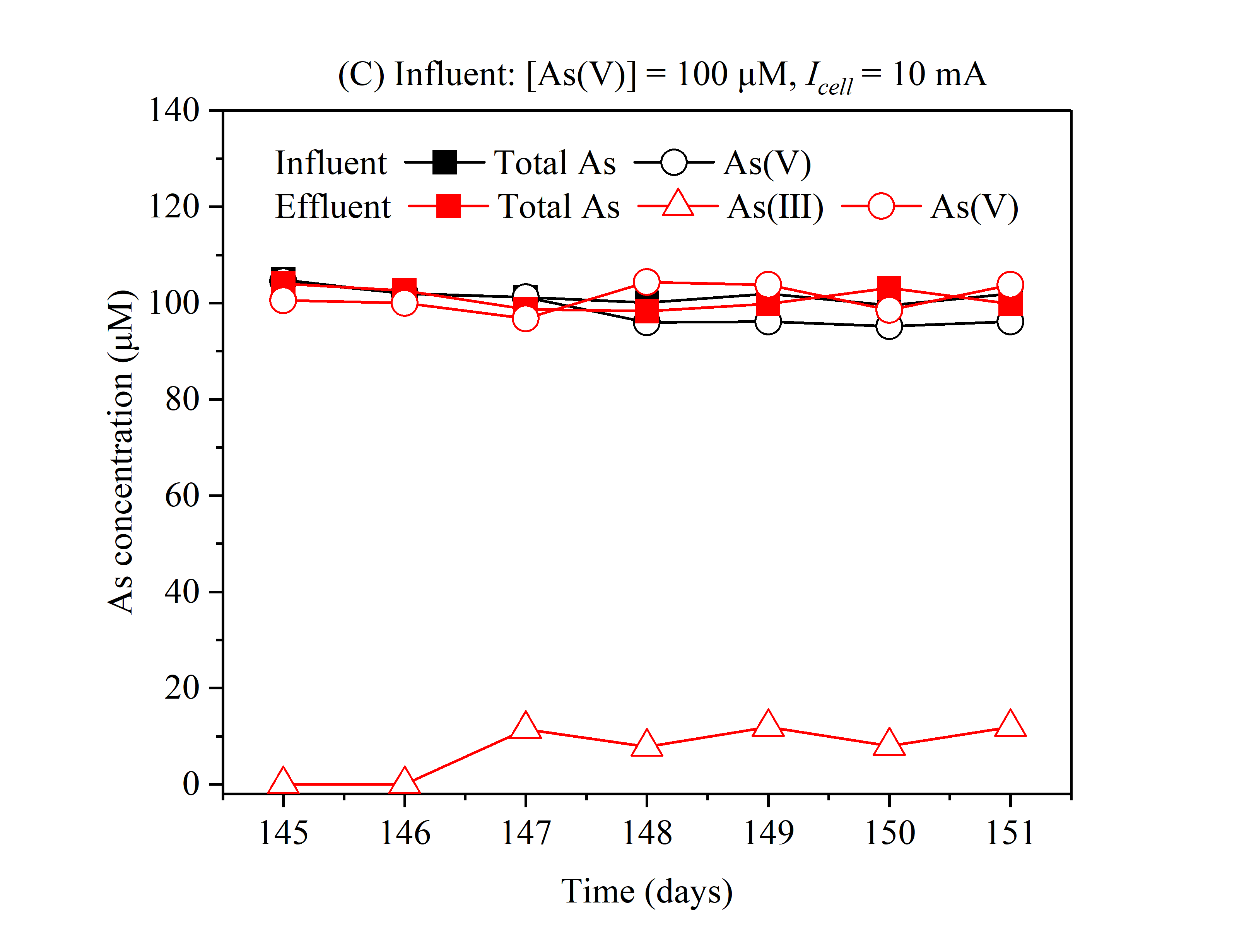


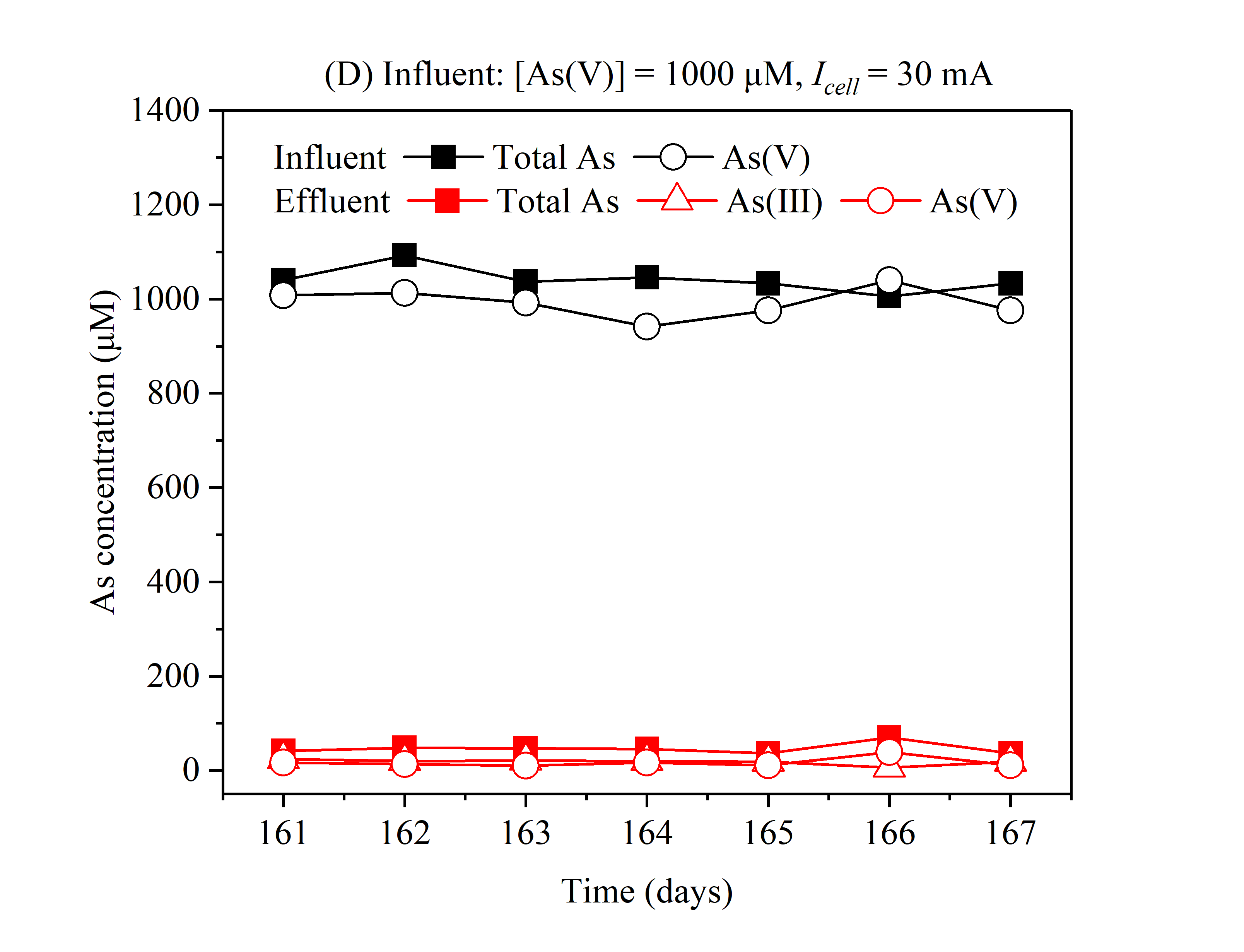

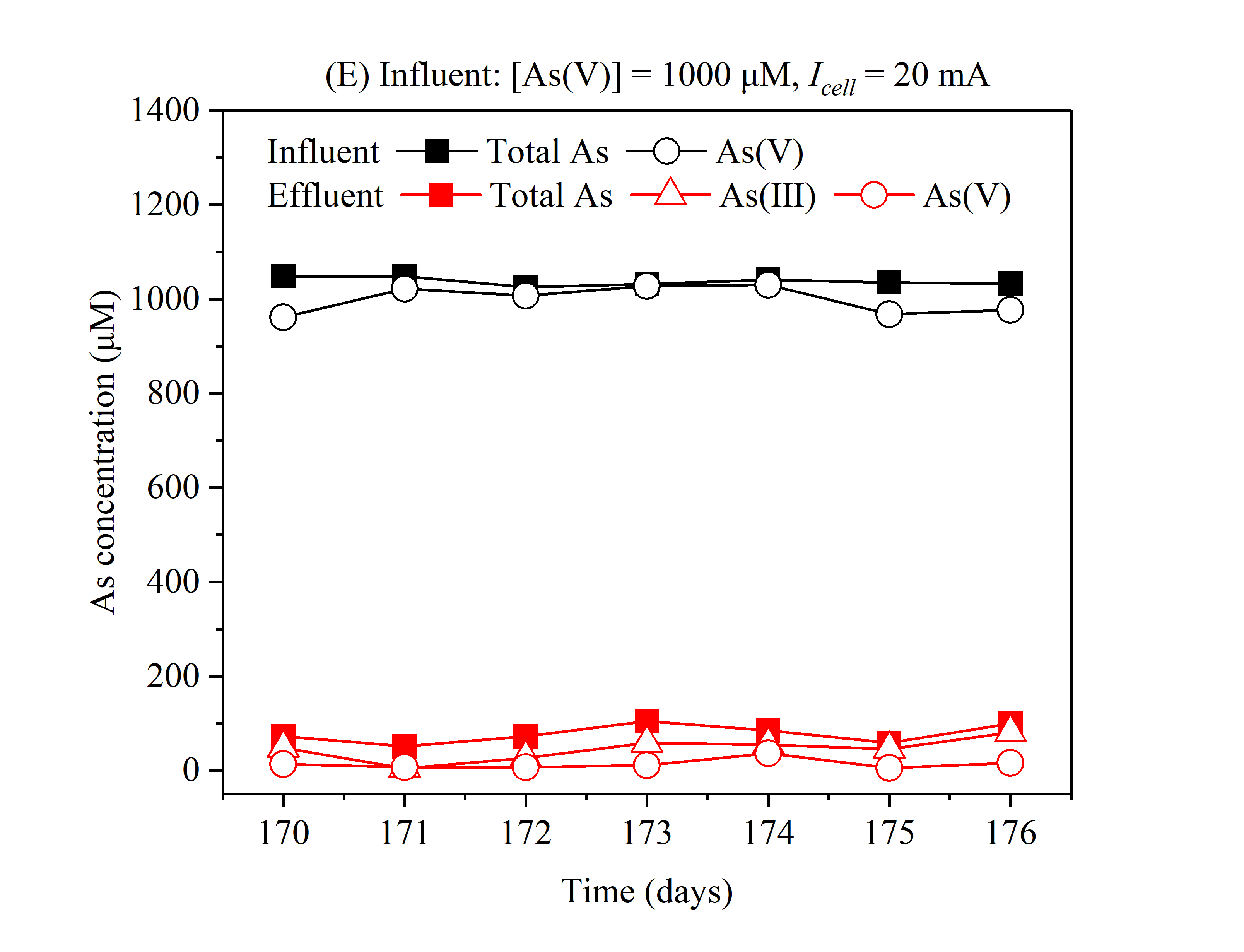

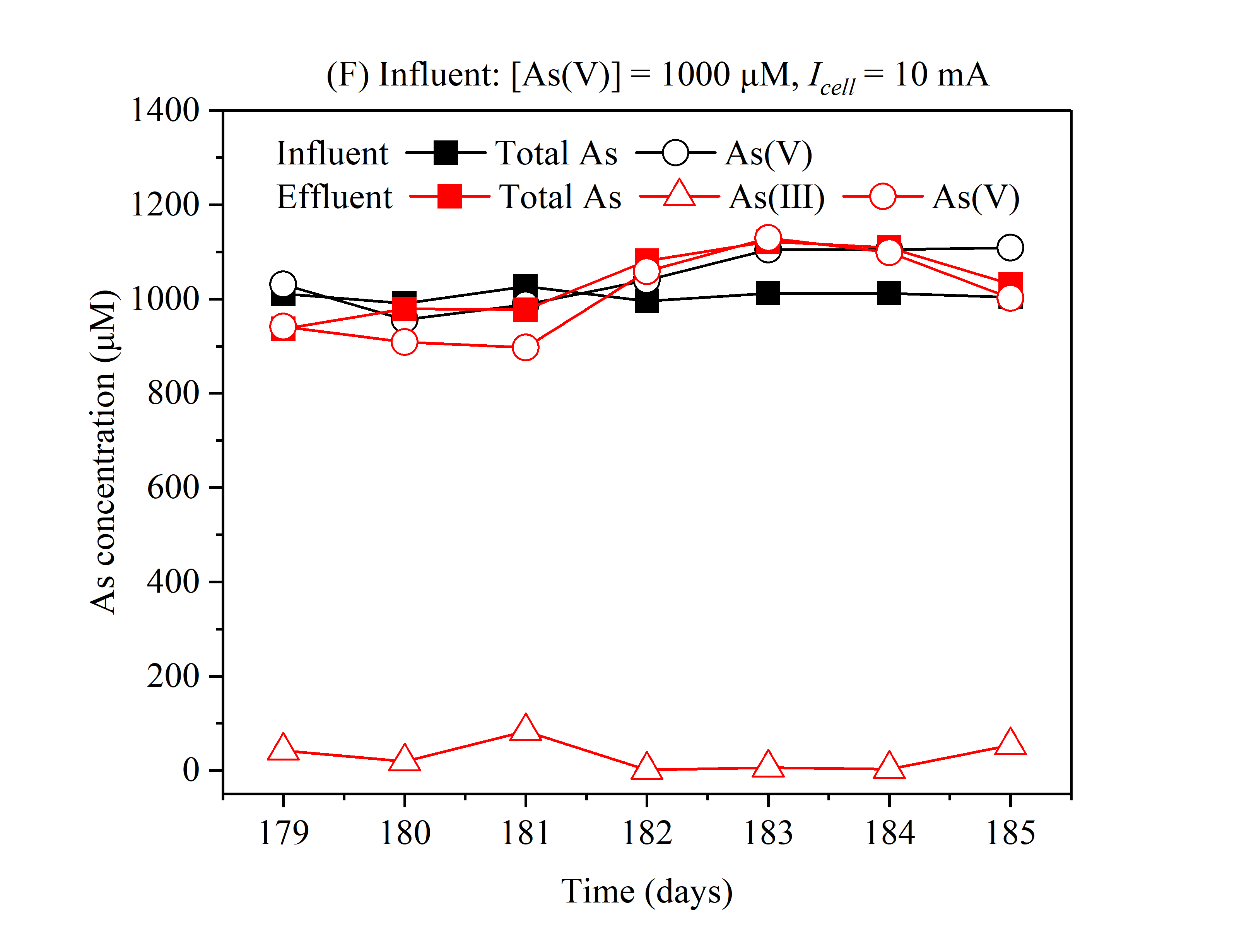
**Figure S2.** Effect of *I_cell_* intensity on As(V) removal in the BES cathode chamber. Experimental conditions: Influent [As(V)] = 100 μM or 1000 μM , [Na_2_SO_4_] = 3.3 mM. The 30 mA, 20 mA and 10 mA of *I_cell_* were applied, respectively.


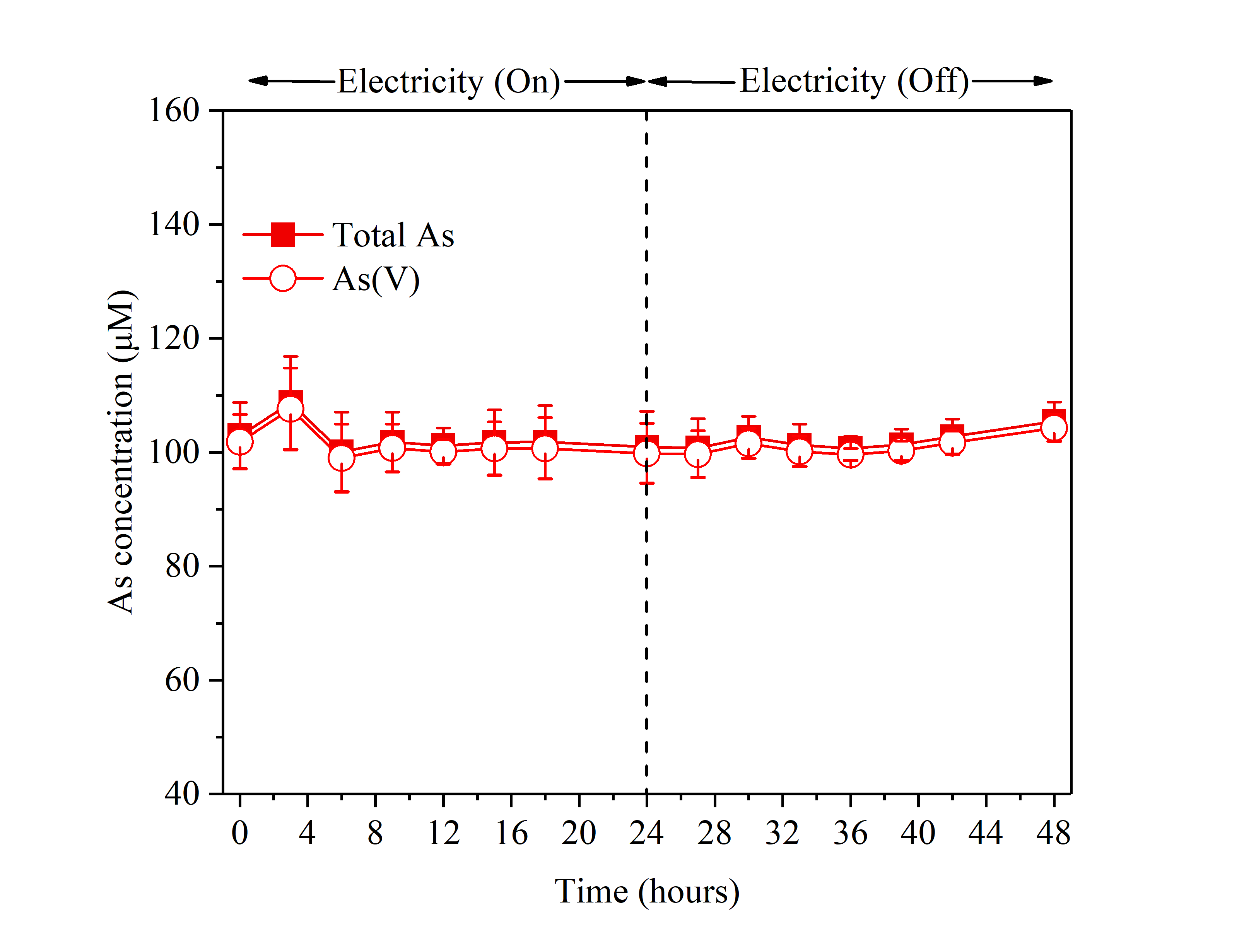


**Figure S3.** Effect of electricity on As(Ⅴ) removal in the abiotic control reactor. Experimental conditions: Influent [As(Ⅴ)] = 100 μM, [Na_2_SO_4_] = 3.3 mM, *I_cell_* = 30 mA. ‘On’ indicates the *I_cell_* was applied, ‘Off’ indicates the *I_cell_* was absent. The error bars show the standard deviation (n = 3).


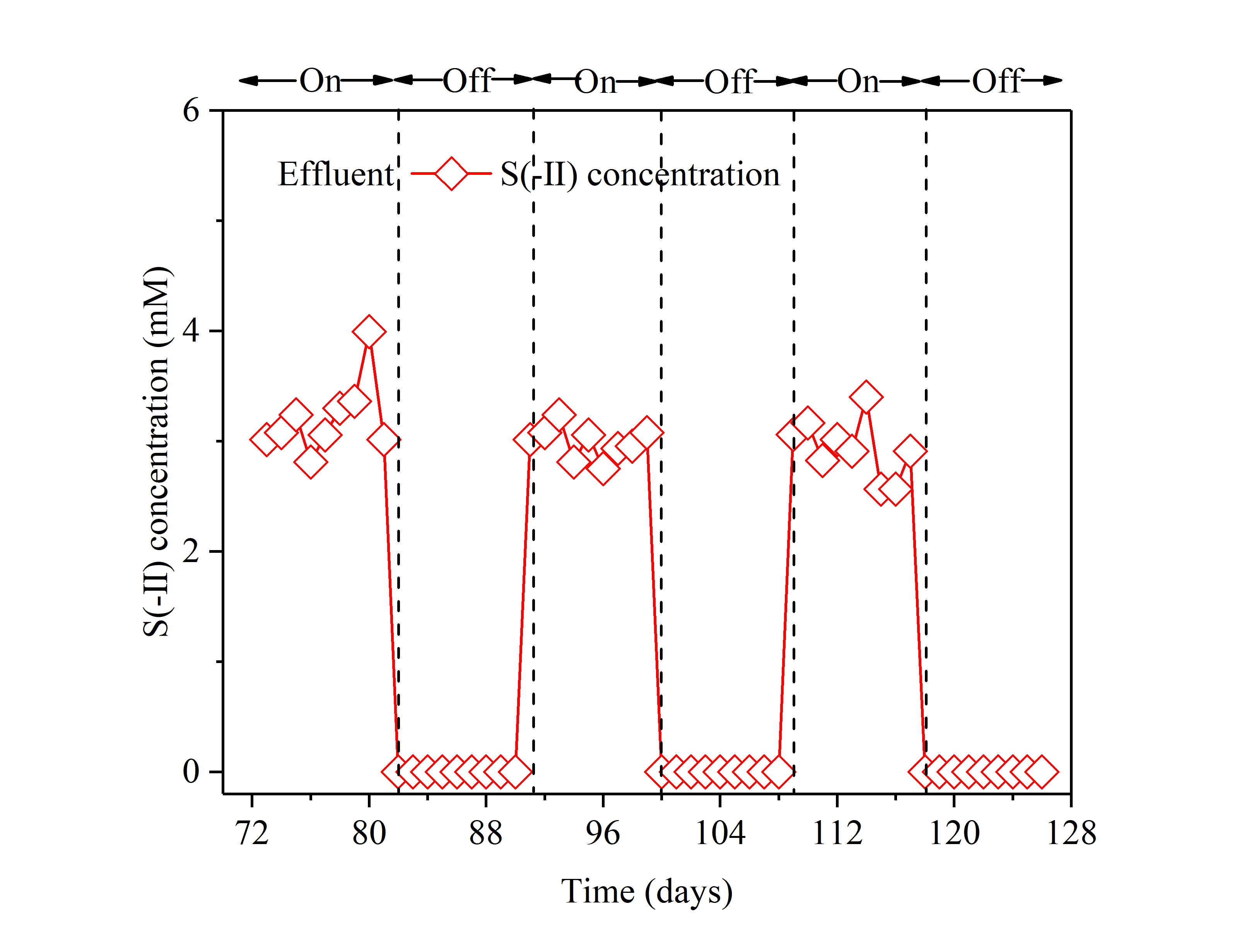


**Figure S4.** Effect of electricity on S(−Ⅱ) concentrations in the BES cathode chamber. Experimental conditions: Influent [As(Ⅴ)] = 100 μM, [Na_2_SO_4_] = 3.3 mM, *I_cell_* = 30 mA. ‘On’ indicates the *I_cell_* was applied, ‘Off’ indicates the *I_cell_* was absent.







**Figure S5.** Scanning electron micrograph of the sludge in the BES cathode chamber. The images were taken using an SE detector at 3.0 kV and 8.3 ± 2.3 mm WD.


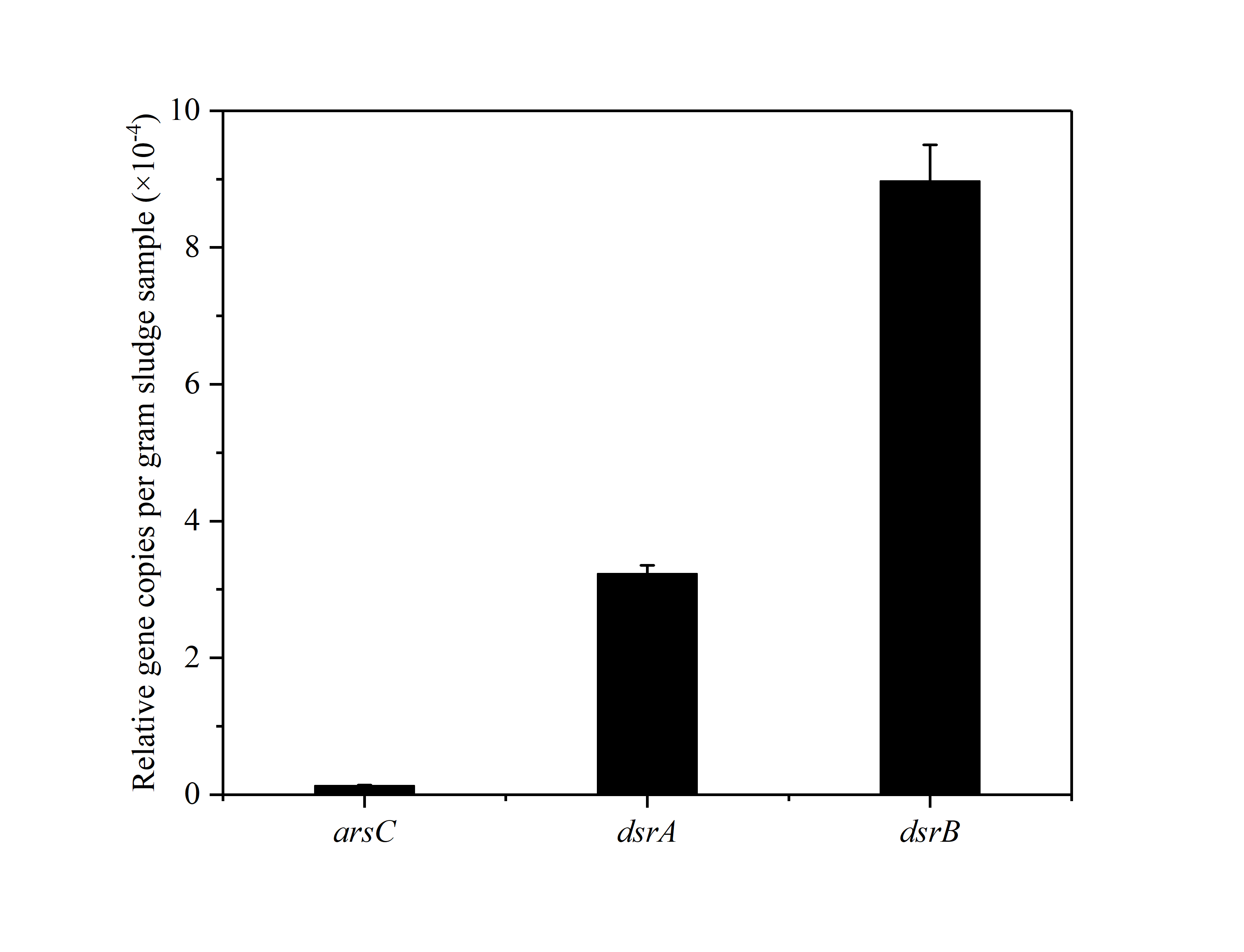


**Figure S6.** Normalized relative gene abundances of *arsC*, *dsrA* and *dsrB* genes in sludge in the BES cathode chamber. The error bars show the standard deviation (n = 3).


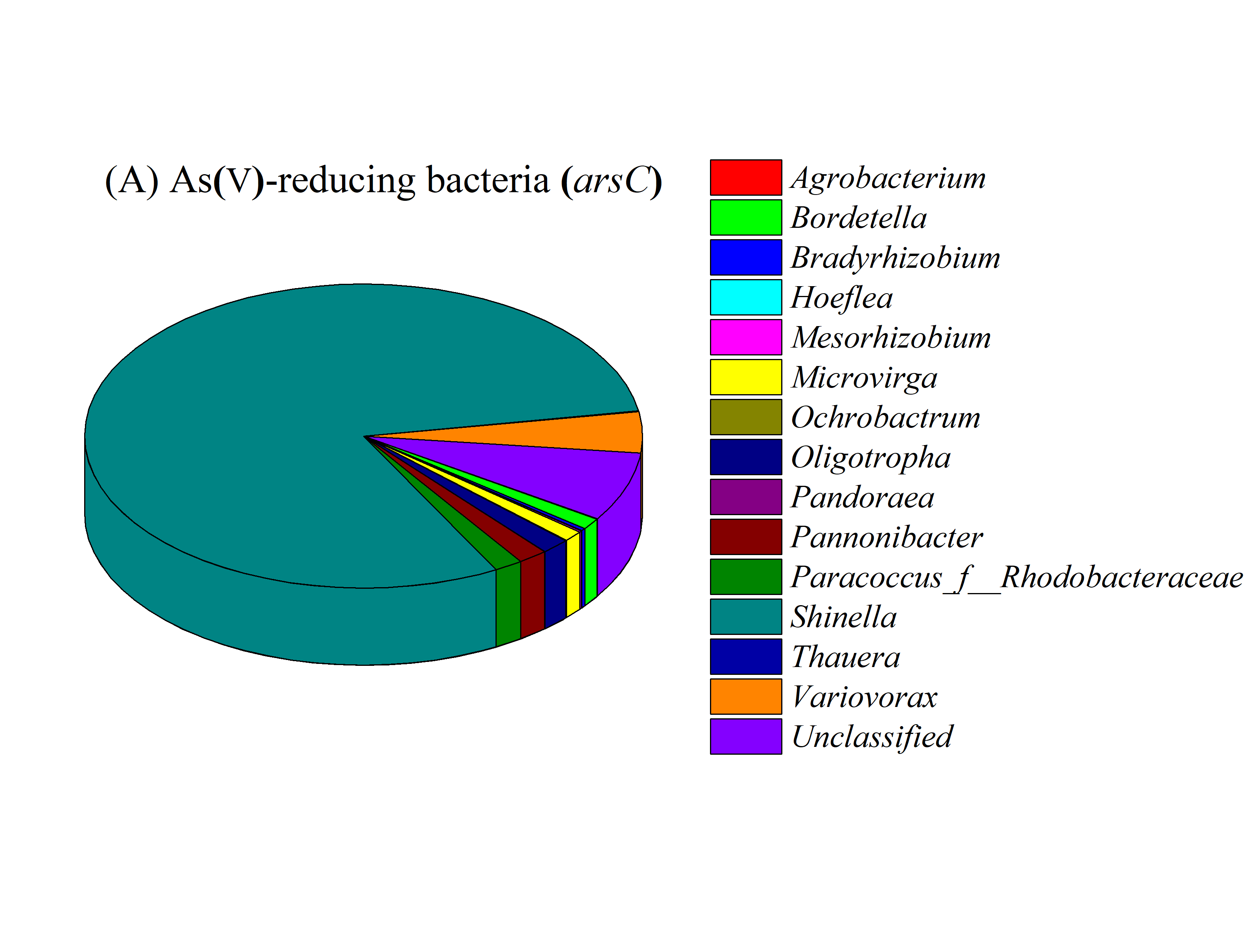


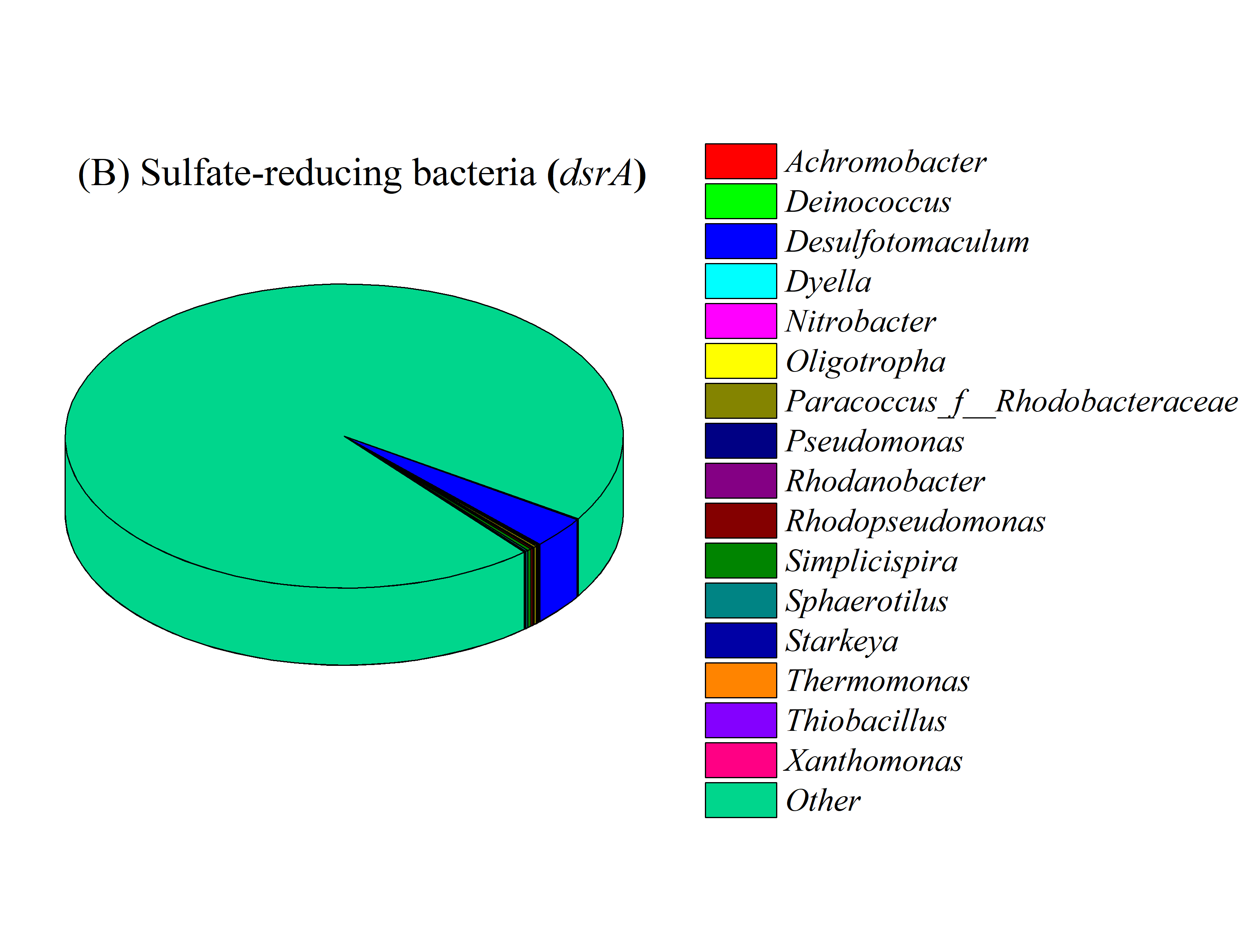


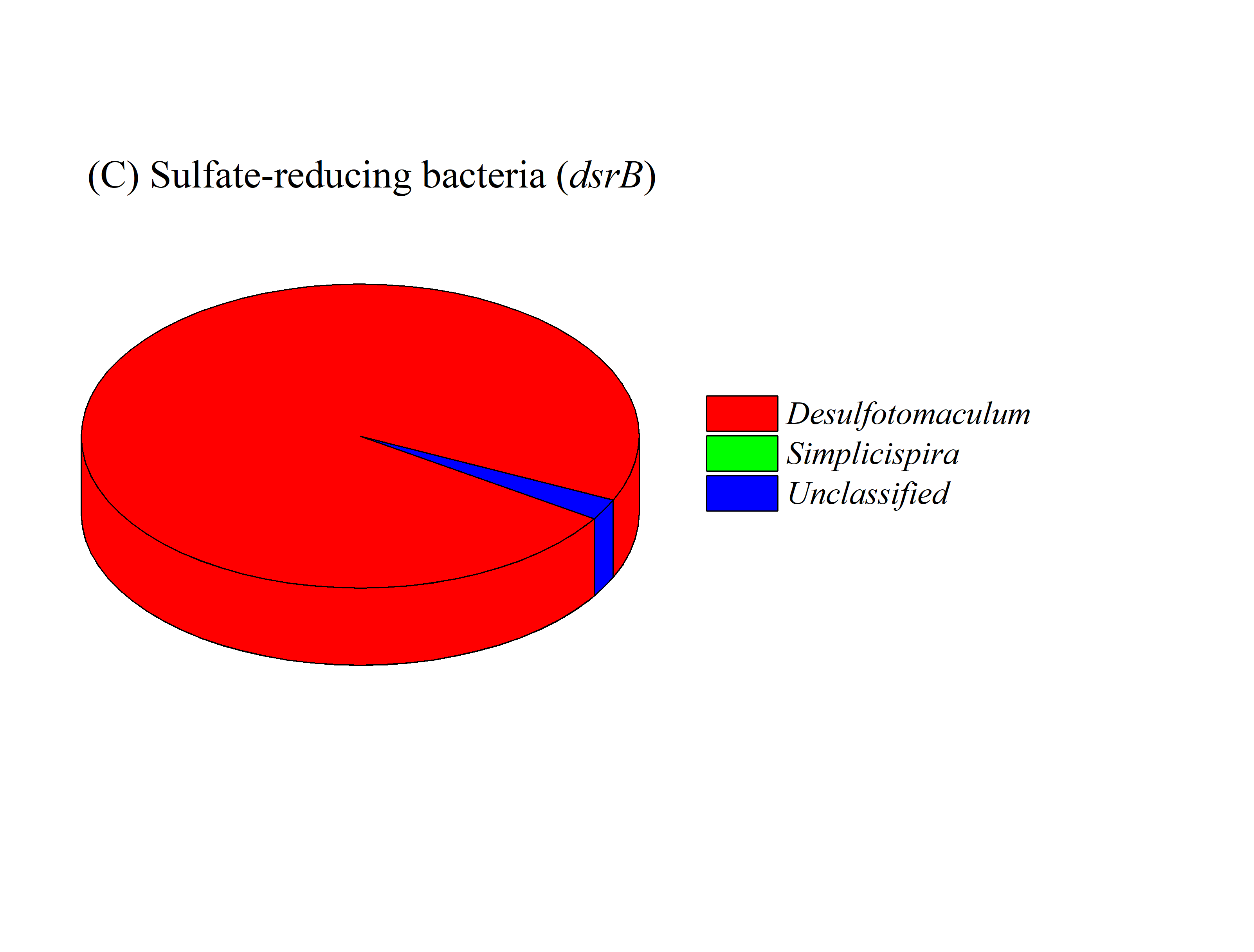


**Figure S7.** Microbial composition of As(Ⅴ)-reducing bacteria encoded by *arsC* gene (A), sulfate-reducing bacteria encoded by *dsrA* gene (B) and *dsrB* gene (C) in the sludge at the genus level. The proportions of reads in each genus for the sludge were shown in Table S4.


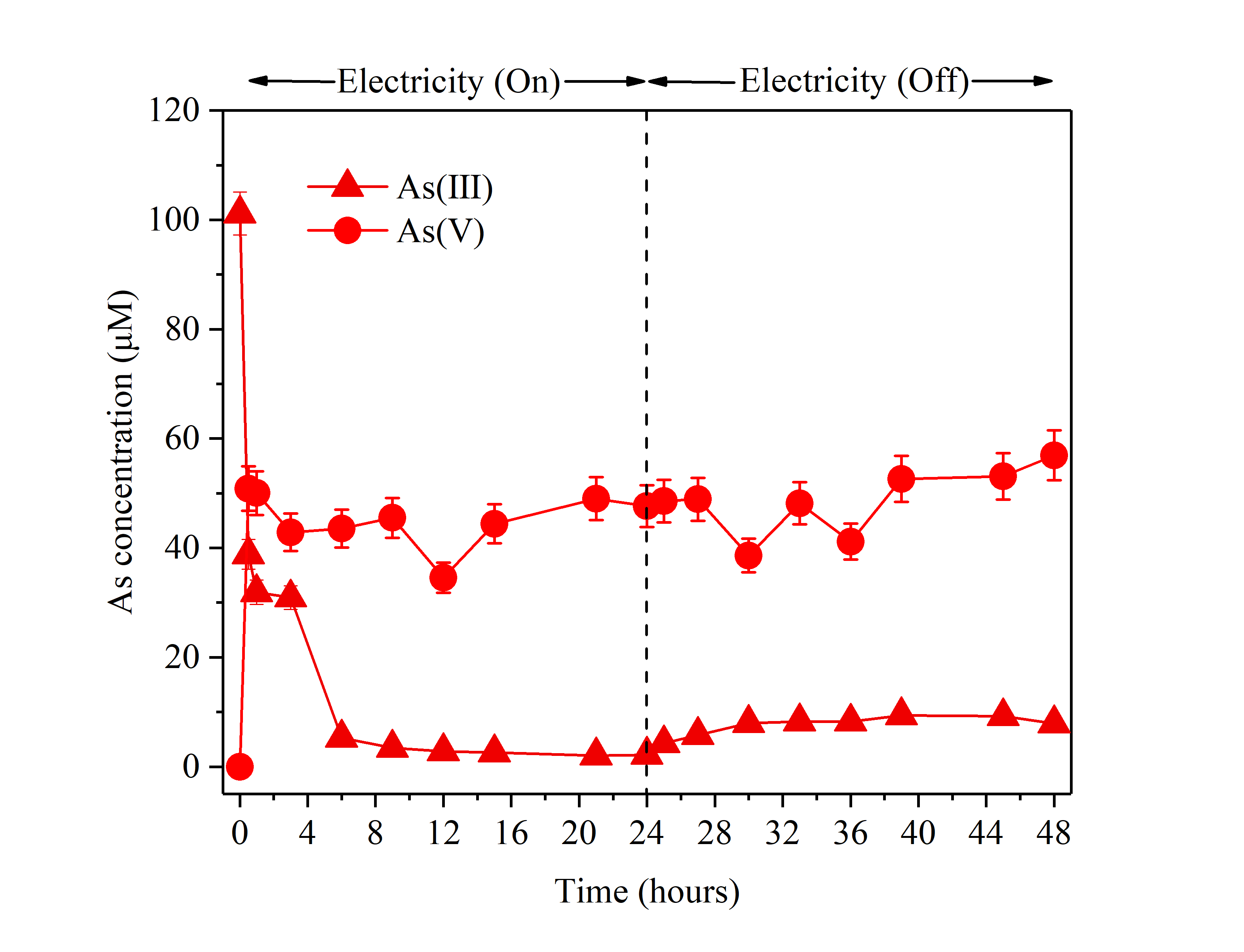


**Figure S8.** Effect of electricity on As(Ⅲ) removal in the abiotic control reactor. Experimental conditions: [As(Ⅲ)] = 100 μM, [Na_2_SO_4_] = 3.3 mM, *I_cell_* = 30 mA. ‘On’ indicates the *I_cell_* was applied, ‘Off’ indicates the *I_cell_* was absent. The error bars show the standard deviation (n = 3).

**References**

Geets, J., Borrernans, B., Diels, L., Springael, D., Vangronsveld, J., van der Lelie, et al. (2006). DsrB gene-based DGGE for community and diversity surveys of sulfate-reducing bacteria. *J Microbiol. Meth.* 66: 194-205. doi:10.1016/j.mimet.2005. 11.002

Gilhotra, V., Das, L., Sharma, A., Kang, T. S., Singh, P., Dhuria, R. S., et al. (2018). Electrocoagulation technology for high strength arsenic wastewater: Process optimization and mechanistic study. *J Clean. Prod.* 198: 693-703. doi:10.1016/j.jclep ro.2018.07.023

Karakurt, S., Pehlivan, E., Karakurt, S. (2019). Removal of carcinogenic arsenic from drinking Water by the application of ion exchange resins. *Oncogen* 2. doi:10.35702/o nc.10005

Kondo, R., Nedwell, D. B., Purdy, K. J., Silva, S. D. (2004). Detection and enumeration of sulphate-reducing bacteria in estuarine sediments by competitive PCR. *Geomicrobiol. J* 21: 145-157. doi:10.1080/01490450490275307

Senn, A.C., Hug, S. J., Kaegi, R., Hering, J.G., Voegelin, A. (2018). Arsenate co-precipitation with Fe(II) oxidation products and retention or release during precipitate aging. *Water Res.* 131: 334-345. doi:10.1016/j.watres.2017.12.038

Sun, Y. M., Polishchuk, E. A., Radoja, U., Cullen, W. R. (2004). Identification and quantification of arsC genes in environmental samples by using real-time PCR. *J Microbiol. Meth.* 58: 335-349. doi:10.1016/j.mimet. 2004.04.015

Suzuki, M. T., Taylor, L. T., DeLong, E. F. (2000). Quantitative analysis of small-subunit rRNA genes in mixed microbial populations via 5'-nuclease assays. *Appl. Environ. Microb.* 66: 4605-4614. doi: 10.1128 /AEM.66.11.4605-4614.2000

Ungureanu, G., Santos, S., Boaventura, R., Botelho, C. (2015). Arsenic and antimony in water and wastewater: Overview of removal techniques with special reference to latest advances in adsorption. *J Environ. Manage.* 151: 326-342. doi:10.1016/j.jenvm an.2014.12.051

Zhang, Q. L., Lin, Y. C., Chen, X., Gao, N. Y. (2007). A method for preparing ferric activated carbon composites adsorbents to remove arsenic from drinking water. *J Hazard. Mater.* 148: 671-678. doi:10.1016/j.jhazmat. 2007.03.026

Zhang, S. Y., Zhao, F. J., Sun, G. X., Su, J. Q., Yang, X. R., Li, H., et al. (2015). Diversity and abundance of arsenic biotransformation genes in paddy soils from southern China. *Environ. Sci. Technol.* 49: 4138-4146. doi:10.1021/acs.est.5b00028

Zhang, Z., Zhu, J. (2006). Characteristics of solids, BOD_5_ and VFAs in liquid swine manure treated by short-term low-intensity aeration for long-term storage. *Bioresource Technol.* 97: 140-149. doi:10.1016/j.biortech.2005.02.002
